# Essential updates 2020/2021: Colorectal diseases (benign)—Current topics in the surgical and medical treatment of benign colorectal diseases

**DOI:** 10.1002/ags3.12548

**Published:** 2022-01-25

**Authors:** Hiroshi Sawayama, Yuji Miyamoto, Naoya Yoshida, Hideo Baba

**Affiliations:** ^1^ Department of Gastroenterological Surgery Graduate School of Medical Sciences Kumamoto University Kumamoto Japan

**Keywords:** Crohn's disease, diverticulitis, inflammatory bowel disease, surgery, ulcerative colitis

## Abstract

The development of new drugs for inflammatory bowel disease (IBD) is remarkable, and treatment strategies using multiple agents and various techniques are required; however, the treatment strategy is likely to be complex. Therefore, appropriate evaluation of traditional surgical treatment strategies and accurate knowledge of the efficacy and limitations of novel treatments are required. Total infectious complications were found to be associated with the use of corticosteroids and anti‐tumor necrosis factor‐α agents, but not with immunomodulators, anti‐integrin agents, and 5‐aminosalicylic acid. Regarding surgical procedures for IBD, conceived anastomosis methods, including Kono‐S for Crohn's disease stenosis, are associated with better outcomes than conventional techniques. Autologous cell transplantation for Crohn's fistulae has been shown to have a favorable outcome. Diverticulitis is increasing and will be treated more frequently in the future. Risk factors for the incidence of diverticulitis and differences in pathogenesis according to right or left side diverticulitis have been reported. Antibiotic therapy may be omitted for uncomplicated diverticulitis. Moreover, regarding surgical procedures, both bowel resection and anastomosis are associated with favorable short‐term outcomes, higher stoma closure rate, and superior medical economy compared to Hartmann's procedure. Risk factors for recurrence after diverticulitis surgery may provide better postoperative follow‐up. In this review, we explore the current topics of colorectal benign diseases, focusing on IBD and diverticulitis, based on clinical trials and meta‐analyses from 2020‐2021. This review consolidates the available knowledge and improves the quality of surgical procedures and perioperative management for IBD and diverticulitis.

## Graphical abstract

In this review, we explore the current topics of colorectal benign diseases, focusing on inflammatory bowel disease and diverticulitis, based on clinical trials and meta‐analyses from 2020 to 2021. This review consolidates the available knowledge and improves the quality of surgical procedures and perioperative management for inflammatory bowel disease and diverticulitis.

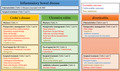

## 1 INTRODUCTION

Inflammatory bowel disease (IBD) and diverticular disease are treated with a combination of medical and surgical therapy. The treatment and perioperative management of IBD and diverticulitis are major concerns among surgeons owing to the frequency and complexity of treatment and the severity of these diseases in clinical practice.

Currently, there are more than 1 million patients with IBD in the United States and 2.5 million in Europe, with substantial costs for health care.^1^ Ulcerative colitis (UC) and Crohn's disease (CD) are two of the most common types of IBD. Infliximab was approved for the treatment of UC by the Food and Drug Administration in 2005 in the United States. Although the number of patients hospitalized for ulcerative colitis increased by over 70% in a nearly linear trend, the rate of patients who underwent total proctocolectomy decreased from 111.1 to 77.1 per 1000 UC admissions between 2002 and 2013, according to the Nationwide Inpatient Sample database. However, the majority of total proctocolectomies (69%) were performed within 24 hours of hospital admission.^2^ The development of recent medical therapeutic agents for IBD has been remarkable; however, in many cases, surgical treatment is still required.

Diverticulitis and diverticular bleeding require medical treatment for colon diverticulosis. Moreover, diverticular disease‐related mortality increased in 58 nations from 1994 to 2016; during this period, the relevant mortality rate increased in 57% of nations, whereas it decreased in only 7% Mortality associated with diverticular disease is increasing worldwide.^3^ Surgical treatment for diverticulitis is likely to increase in the future.

**FIGURE 1:**
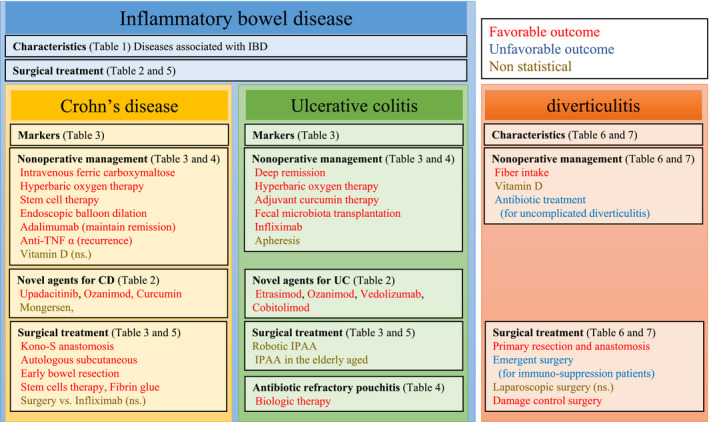
Summary of key articles on clinical trials and meta‐analyses of inflammatory bowel disease and diverticulitis

Benign colorectal disease from 2018 to 2019 was reviewed in this journal.^4^ Several clinical trials and meta‐analyses have revealed novel treatment strategies and outcomes of these treatments developed in 2020. In the present review, newly determined characteristics, prognostic markers, non‐operative management, and surgical treatment strategies optimal for IBD and diverticulitis are reviewed in accordance with articles published in the last 2 years (2020‐2021) (Figure 1).

## 2 IBD

### 2.1 Association between IBD and other diseases

Arthritis, uveitis, pancreatitis, primary sclerosing cholangitis, and erythema nodosum are extraintestinal complications associated with IBD. Other diseases accompanying IBD have been reported in previous meta‐analyses (Table 1). Inflammatory resorption of alveolar bones is caused by polymicrobial biofilm‐mediated disease. Moreover, inflammatory processes are similar in periodontitis and IBD; the presence of periodontitis was associated with IBD, and periodontitis was strongly associated with both CD and UC.^5^ The prevalence of cutaneous symptoms, pyoderma gangrenosum, psoriasis, and herpes zoster infection was frequently revealed to be accompanied by IBD.^6^, ^7^ Patients with IBD had an increased risk of infection due to immune system dysregulation associated with the use of corticosteroids, immunosuppressant drugs, and anti‐ tumor necrosis factor (TNF)α. The risk of herpes zoster infection increased in CD (Risk ratio [RR]: 1.74, steroid users RR: 1.78) and UC (RR: 1.40, steroid users RR: 1.99, anti‐TNFα users RR: 2.29). Patients with IBD with a high risk of HZ infection may benefit from an HZ vaccine.^8^ Skin lesions are likely to be associated with IBD, and when treating patients with IBD, it is important to carefully examine the skin condition.

**TABLE 1:** Meta‐analyses of characteristics and markers inflammatory bowel disease

Focus	Main results	Reference
Periodontitis	The association between periodontitis and UC: present (OR 5.37)	*BMC Oral Health*. 2020 Mar 12;20(1):67
Periodontitis	The relation between periodontitis and IBD: OR: 2.10 (CD 1.72, UC: 2.39)	Biomed Res Int. 2021 Mar 12;2021:6692420
Periodontal disease (PD)	The presence of PD was associated with IBD: OR: 2.78 (CD: 3.41, UC 3.98)	*Acta Odontol Scand*. 2021 Jul;79(5):344‐35
Pyoderma gangrenosum (PG)	The incidence of PG in IBD: 0.4 to 2.6%. PG was associated with female gender (RR: 1.33), CD (RR: 1.19), erythema nodosum (RR: 9.28), and ocular extra‐intestinal manifestation (RR: 4.55)	Dig Dis Sci. 2020 Sep;65(9):2675‐2685
Psoriasis	The prevalence of psoriasis: CD 3.6% and UC 2.8%.	*J Crohns Colitis*. 2020 Mar 13;14(3):351‐360
Herpes zoster (HZ) infection	Risk of HZ infection: CD: RR: 1.74 (steroid users RR: 1.78). UC: RR: 1.40 (steroid users RR: 1.99, anti‐TNFα users RR: 2.29) IBD patients with high risk of HZ infection may benefit from an HZ vaccine	*Eur J Clin Microbiol Infect Dis*. 2020 Feb; 39(2):219‐227
Rheumatoid arthritis	The association between IBD and the risk of RA: higher risk of RA among patients with IBD: RR 2.59 (CD, RR: 3.14, UC, RR: 2.29)	*BMC Gastroenterol*. 2020 Jun 17;20(1):192
Elderly onset (EO) and adult onset (AO) IBD	EO = AO: 5‐year risk of surgery, overall exposure to corticosteroids EO < AO: exposure to immunomodulators, biologic agents	*Clin Gastroenterol Hepatol*. 2020 Oct;18(11):2437‐2447
Anxiety and depression	Anxiety symptoms: 32.1%, depression symptoms: 25.2% CD > UC: anxiety symptoms (OR 1.2), depression symptoms (OR 1.2)	*Lancet Gastroenterol Hepatol*. 2021 May;6(5):359‐370
Opioid use	Prevalence of opioid use: outpatients setting 21%, hospitalized 62% female (RR 1.20), depression (RR 1.99), substance abuse (RR 4.67), prior surgery (RR 2.33), biologic use (RR 1.36), steroid use (RR 1.41)	*Clin Gastroenterol Hepatol*. 2021 May;19(5):895‐907.e4.

Anxiety and depression are commonly experienced by patients with IBD. The prevalence of corresponding symptoms was 32.1% and 25.2%, respectively, and the incidence of such was higher in patients with CD than in those with UC.^9^ Moreover, these symptoms were associated with CD‐related surgery, the Crohn's disease activity index, and corticosteroid use in patients with CD.^10^ Patients with IBD often experience anxiety and depression during the perioperative period, and appropriate psychological care may be beneficial in these patients.

### 2.2 Novel agents for IBD

The development of novel therapeutics for IBD is remarkable, so much so that it is difficult for general surgeons to understand all of them. The perioperative impact of these drugs will be discussed later; here, we enumerate novel therapeutic agents that were reported from 2020 to 2021. The results of the clinical trials are summarized in Table 2. Table S1 presents a summary of the approval status of novel agents.

**TABLE 2:** Novel agents for inflammatory bowel disease

Agents	Target	Endpoints	Main results	Reference
Crohn's disease				
Upadacitinib	Janus kinase 1 inhibitor	Clinical remission (16 wk) and endoscopic remission (12 wk)	Endoscopic (not clinical) remission increased	*Gastroenterology*. 2020 Jun;158(8):2123‐2138.e8
Ozanimod	Sphingosine‐1‐phosphate receptor subtypes 1 and 5	Endoscopic Score for Crohn's Disease (SES‐CD) from baseline to 12 wk	The mean change SES‐CD: 2.2, endoscopic, histological, clinical improvements	*Lancet Gastroenterol Hepatol*. 2020 Sep;5(9):819‐828
Mongersen	Antisense oligodeoxynucleotide to Smad7	Clinical remission CD Activity Index score <150 (12 wk)	NS	*Am J Gastroenterol*. 2020 May;115(5):738‐745
Ulcerative colitis				
Etrasimod	Sphingosine 1‐phosphate receptor modulator	Improvement in modified MCS (etrasimod vs placebo)	Etrasimod favorable (*P* = 0.009) endoscopic improvement: 41.8% vs 17.8%	*Gastroenterology*. 2020 Feb;158(3):550‐561
Ozanimod	Sphingosine‐1‐phosphate receptor subtypes 1 and 5	Induction and maintenance therapy (ozanimod vs placebo)	Clinical remission (18.4% vs 6.0%, *P* < .001), maintenance (37.0% vs 18.5%, *P* < .001)	*N Engl J Med*. 2021 Sep 30;385(14):1280‐1291
Vedolizumab	Inhibits the gut‐selective α4β7 integrin	Maintenance treatment, intravenous vedolizumab vs placebo groups	Clinical remission: 46.2%, 42.6%, and 14.3%, respectively	*Gastroenterology*. 2020 Feb;158(3):562‐572
Cobitolimod	Activates Toll‐like receptor 9	The proportion of clinical remission	Cobitolimod: 21% vs placebo: 7% (OR 3.8)	*Lancet Gastroenterol Hepatol*. 2020 Dec;5(12):1063‐1075

The effects and tolerability of upadacitinib (a selective Janus kinase 1 inhibitor),^11^ ozanimod (targeting sphingosine‐1‐phosphate receptor subtypes 1 and 5),^12^ and mongersen (antisense oligodeoxynucleotide to Smad7)^13^ have been reported in clinical trials for CD.

Novel agents for UC, including etrasimod (selective sphingosine 1‐phosphate receptor modulator),^14^ ozanimod,^15^ vedolizumab (inhibits the gut‐selective α4β7 integrin),^16^ cobitolimod (activates Toll‐like receptor 9),^17^ and budesonide (systemic corticosteroids)^18^ were reported from 2020 to 2021.

A meta‐analysis showed that anti‐TNF‐α agents prevented endoscopic recurrence (RR: 0.34) but not clinical recurrence (RR: 0.60, not significant [n.s.]). Patients receiving anti‐TNFα therapy experienced more adverse effects than those who were not (RR: 1.75).^19^ Curcumin, as an adjuvant treatment for mesalamine, was proven to be effective in inducing clinical remission (odds ratio [OR]: 5.2), endoscopic remission (OR: 5.7), and endoscopic improvement (OR: 17.1), and was shown to be safe in UC.^20^

### 2.3 Non‐drug treatment for IBD

Hyperbaric oxygen (HBO) and endoscopic balloon dilation are typical treatments for bowel obstruction caused by adhesion and stenosis, respectively. The favorable effects of these treatments on the symptoms associated with IBD are described below (Table 3 and 4).

**TABLE 3:** Clinical trials of inflammatory bowel disease

Factor	Endpoints	Main results	Reference
Markers of inflammatory bowel disease			
Disease activity miR‐320a levels	Appropriate clinical disease indices and endoscopic scoring systems	MiR‐320a expression (peripheral blood) are associated with the clinical and endoscopic disease activities of IBD	*Clin Transl Gastroenterol*. 2020 Mar;11(3):e00134
Response to infliximab in CD	Predicting mucosal healing	Oncostatin M can predict the outcome of infliximab treatment. (AUC = 0.91)	*Aliment Pharmacol Ther*. 2020 Jul;52(2):284‐291
Vedolizumab	Variables response to vedolizumab (against the α4β7 integrin heterodimer)	Markers associated with vedolizumab ‐induced clinical remission: CD: IL17A, TNF, CXCL1, CCL19, UC: G‐CSF and IL7	*Clin Gastroenterol Hepatol*. 2021 Mar;19(3):503‐510.e1
Anxiety/depression	The risk factors of anxiety/depression in IBD	CD: CD‐related surgery and CDAI/depression in IBD UC: corticosteroid use	*Sci Rep*. 2021 Jan 14;11(1):1440
Microbial factors	Maintenance of remission	Favorable: Lac unfavorable: Enterobacteriaceae (OR 6.35) Lachnospiraceae family (OR 0.47)	*Gut Microbes*. 2020 Nov 1;11(6):1713‐1728
Faecal calprotectin (Fcal)	Prediction of postoperative recurrence after ileocolonic resection (CD)	Fcal variation: predictor of early endoscopic postoperative recurrence AUC = 0.73, sensitivity = 64.7%, specificity = 87.5%	*Dig Liver Dis*. 2020 Jul;52(7):740‐744
Nonoperative treatment for IBD			
HBO for chronic antibiotic‐refractory pouchitis (CARP)	The efficacy and safety of HBO for CARP	mPDAI symptom score: 3.19 to 1.91 mPDAI endoscopy scores: 2.34 to 1.29 improving CARP	*Inflamm Bowel Dis*. 2021 Jun 15;27(7):965‐970
Nonoperative treatment for Crohn's disease			
Oral Sucrosomial^®^ Iron	Intravenous ferric carboxy ‐maltose (FMC) vs Sucrosomial^®^ Iron (SI)	FCM = SI: Hemoglobin, Iron (4, 8, 12 wk) FCM > SI: Ferritin levels	*Nutrients*. 2021 Feb 12;13(2):608
Vitamin D	Endoscopic recurrence (26 wk)	Vitamin D vs placebo (58% vs 66%, NS)	*Clin Gastroenterol Hepatol*. 2021 Aug;19(8):1573‐1582.e5
Hyperbaric oxygen therapy (HBO)	Efficacy, safety, and feasibility of HBO in CD	Clinical response: 60% Clinical remission: 20%	*Aliment Pharmacol Ther*. 2021 Mar;53(5):587‐597
Nonoperative treatment for ulcerative colitis			
Deep remission (a tight control strategy)	Major adverse outcomes that indicate CD progression	Favorable: deep remission CD endoscopic index of severity scores below 4, with no deep ulcerations or steroid use	*Gastroenterology*. 2020 Jul;159(1):139‐147
HBO 2.4 atmospheres (90 min)	Day 3 responders: 5 days vs 3 days of HBO	HBO favorable: low rates of re‐hospitalization, colectomy at 3 mo (0% vs 66%)	*Aliment Pharmacol Ther*. 2020 Sep;52(6):955‐963
Fecal microbiota transplantation (FMT)	T regulatory and mucosal associated invariant T (MAIT) cell populations	Changes in MAIT cell cytokine production were observed in cFMT	*BMC Gastroenterol*. 2021 Jul 8;21(1):281
Apheresis selective removal of leukocytes	Clinical remission (Mayo score ≤2) at 12 mo	Apheresis: 46.6%, control: 36.4% (NS)	*J Gastroenterol*. 2020 Apr;55(4):390‐400
Cannabis 80 mg tetra ‐hydrocannabinol	Lichtiger disease activity index, CRP, calprotectin, Mayo endoscopic score and QOL	Cannabis favorable: Lichtiger index, QOL Cannabis = placebo: Mayo endoscopic score	*PLoS One*. 2021 Feb 11;16(2):e0246871
Surgical treatment for Crohn's disease			
Surgery vs Infliximab	Need for surgery or repeat surgery or anti‐TNF therapy	Treatment effect was similar Laparoscopic ileocecal resection: not successful	*Lancet Gastroenterol Hepatol*. 2020 Oct;5(10):900‐907
Kono‐S anastomotic methods	Kono‐S vs stapled ileocolic side‐to‐side anastomosis Endoscopic recurrence (ER)	22.2% in the Kono‐S group and 62.8% in the Conventional group presented an ER	*Ann Surg*. 2020 Aug;272(2):210‐217
Autologous subcutaneous adipose tissue	Clinical and radiographic healing at 6 mo	All patient: reduction in the size of fistula tracts 3 of 5: cessation of drainage None: complete healing	*Inflamm Bowel Dis*. 2020 Apr 11;26(5):670‐677
Autologous adipose‐derived stem cells	The closure of fistulas at months 3, 6, and 12	Healing rate (3, 6, 12m): the observation vs control, 90.9% vs 45.5%, 72.7% vs 54.5%, and 63.6% vs 54.5%, respectively (NS)	*Stem Cell Res Ther*. 2020 Mar 17;11(1):124
Allogeneic mesenchymal stem cells	Follow‐up 1 y after the procedure	Perianal abscess (15%), complete closure (69%)	*Dis Colon Rectum*. 2021 Mar 1;64(3):328‐334
Allogeneic mesenchymal stromal cells	Fistula closure using bone marrow‐derived mesenchymal stromal cells	Fistulas with closure at 24 wk were still closed after 4 y	*J Crohns Colitis*. 2020 Jan 1;14(1):64‐70
Fibrin glue	The rate of complete clinical remission at 1 y	Complete clinical remission (1 y): 45.4%	*Gastroenterology*. 2021 Feb;160(3):710‐719.e2

Abbreviations: ACU, area under the curve; OR, odds ratio; TNF: tumor necrosis factor.

**TABLE 4:** Meta‐analyses of nonoperative treatment for inflammatory bowel disease

Focus	Endpoints	Main results	Reference
Nonoperative treatment for inflammatory bowel disease			
Sleep quality	The relation between sleep quality and disease activity	Subjective sleep quality and disease activity (OR 3.52), sleep efficiency and disease activity (OR 4.55)	*Sleep Med*. 2020 Nov;75:301‐308
Iron supplementation	Ferric carboxymaltose (FCM), iron isomaltoside (IIM), iron sucrose (IS), oral iron (OI)	Response rates with FCM, IIM, IS, OI: 81%, 74%, 75%, 69% FCM: the most cost‐effective	*Adv Ther*. 2021 Jan;38(1):660‐677
Hyperbaric oxygen therapy (HBO)	Response rate and complete healing of fistula	Response rate of HBO, UC: 83.2%, CD: 81.9%, the complete healing of fistula: 47.6%	*Eur J Gastroenterol Hepatol*. 2021 Apr 19
Antibiotic refractory pouchitis	The safety and efficacy of various biological agents for antibiotic refractory pouchitis	Clinical improvement: IFX 71.4%, ADA 58.2%, VDZ 47.9%, remission: IFX 65.7%, ADA 31%, VDZ 47.4%	*J Clin Gastroenterol*. 2021 Jul 1;55(6):481‐491
Nonoperative treatment for ulcerative colitis			
Adjuvant curcumin therapy	Clinical and endoscopic remission	Clinical remission (OR 5.18) Endoscopic remission (OR 17.05) Clinical improvement (OR 4.79 NS)	*J Gastroenterol Hepatol*. 2020 May;35(5):722‐729
Infliximab (IFX) vs cyclosporine and tacrolimus (TAC)	Short‐term remission, short‐term, colectomy rate	IFX favorable: lower short‐term (OR 0.59), 1 y colectomy rate (OR 0.53), 3 y colectomy rate (OR 0.41)	*Medicine*. 2020 Oct 30;99(44):e22894
Faecal microbiota transplantation (FMT)	Safety and effectiveness of treating UC	Efficacy: FMT favorable (OR 2.29) Multiple donors delivered (OR 2.76) Side effects: NS	*Int J Colorectal Dis*. 2020 Jun;35(6):1025‐1034
Nonoperative treatment for Crohn's disease			
Adalimumab	Failure to maintain clinical remission in people with quiescent CD	Adalimumab: 59% vs placebo: 86% Adalimumab is an effective therapy for maintenance of clinical remission	*Cochrane Database Syst Rev*. 2020 May 16;5(5):CD012877
Anti‐tumor necrosis factor (TNF) α	Preventing endoscopic and clinical recurrence	Endoscopic recurrence (RR 0.34) clinical recurrence (RR 0.60, NS) AEs with anti‐TNF therapy (RR 1.75)	*J Gastroenterol Hepatol*. 2021 Apr;36(4):864‐872
Stem cell therapy	CD activity index	Reduce: CD activity index, CD endoscopic index of severity, simplified endoscopy score for CD	*Stem Cell Res Ther*. 2021 Aug 18;12(1):463
Placebo	The rate of response to placebo endoscopic assessment of CD activity	Response: 16.2%, remission 5.2% lower rates of response to placebo increased concentration of CRP	*Clin Gastroenterol Hepatol*. 2020 May;18(5):1121‐1132.e2
Endoscopic balloon dilation	Small intestinal strictures in CD evaluate endoscopic balloon dilation	Technical success: 94.9%, efficacy: 82.3%, complications: 5.3%, rec.: 48.3% (re‐dilated: 38.8%, surg.: 27.4%)	*Aliment Pharmacol Ther*. 2020 Oct;52(7):1104‐1116

A previous study reported patients with UC who were hospitalized for acute flares and were treated with HBO. The patients who responded to HBO treatment on day 3 required less re‐hospitalization or colectomy than non‐responders (0% vs 66%).^21^ Moreover, patients with CD (n = 20) with high perianal fistula(s) who failed to respond to conventional treatment were treated with HBO, and the rates of clinical response and clinical remission were 60% and 20%, respectively.^22^ In a meta‐analysis, the response rate of HBO was 83.2% in UC and 81.9% in CD, while complete healing of the fistula was noted in 47.6% of fistulizing CD cases.^23^

Endoscopic balloon dilation for small intestinal strictures in CD was evaluated in a previous meta‐analysis. The technical success rate of endoscopic balloon dilation was 94.9%, major complication rate was 5.3%, symptom recurrence rate was 48.3%, and rate of re‐dilation or surgery was 38.8% and 27.4%, respectively.^24^

The safety and efficacy of fecal microbiota transplantation (FMT) for the treatment of *Clostridioides difficile* infections have been reported.^25^ The intestinal flora plays an important role in the progression of UC. FMT has been shown to change the production of mucosal‐associated invariant T cell cytokines.^26^ In a meta‐analysis, the safety and effectiveness of FMT for treating UC was reported.^27^ Reportedly, FMT did not have a sustained effect on the treatment of UC patients unless the administration was repeated and prolonged.^28^ The efficacy of FMT depends on microbial interactions between the donor and recipient strains.^29^ The interactions between bacterial and metabolic pathways are also associated with the induction of remission.^30^ Future studies are needed so that a sustained therapeutic effect can be obtained after FMT treatment in UC patients.

### 2.4 Surgical management for IBD

In patients with CD, stenosis and fistula are the main indications for surgery. The optimal time for surgery and surgical procedures are the main concerns of surgeons (Table 3 and 5). The effects of laparoscopic ileocecal resection and infliximab were similar to those reported in a retrospective study (n = 134).^31^ However, the incidence of relapse in patients with ileocolonic CD (n = 1863) after early bowel resection was compared to that after initial therapy, and the overall (OR: 0.53) and surgical relapse (OR: 0.47) were lower in patients who underwent early bowel resection than in those who received initial medical therapy. Moreover, the requirement for maintenance biologic therapy (OR: 0.24) was lower in patients who received early bowel resection than in those who received initial medical therapy.^32^ The incidence of relapse after strictureplasty was also compared to that of bowel resection for patients with CD. The results demonstrated that strictureplasty alone increased disease recurrence compared to bowel resection (hazard ratio [HR]: 1.61), and the morbidity rate was not significantly different between the two groups.^33^ Antimesenteric cutback end‐to‐end isoperistaltic anastomosis, known as Sasaki‐W anastomosis, has been reported as a novel hand‐sewn anastomotic technique for CD.^34^ In a previous randomized control trial (RCT), Kono‐S anastomosis, antimesenteric functional end‐to‐end handsewn anastomosis, were performed for the stenosis of the patients with CD, and the endoscopic recurrence was 22.2% in the Kono group and 62.8% in the conventional group (n = 79).^35^ In a previous meta‐analysis, the surgical outcomes of Kono‐S were found to be 0% for surgical recurrence and 5% for endoscopic recurrence.^36^ Kono‐S anastomosis yields a favorable outcome with increasing evidence, hence may be considered an optimal procedure for CD stenosis.

**TABLE 5:** Meta‐analyses of surgical treatment for inflammatory bowel disease

Focus	Endpoints	Main Results	Reference
Surgical treatment for inflammatory bowel disease			
Urgent surgeries vs elective surg.	Overall postoperative complications (30d)	Urgent surgery: ~ 40% increase in overall complication (RR: 1.43) Mortality and readmission rates: NS	*Int J Colorectal Dis*. 2021 Feb;36(2):253‐263
Robotic vs laparoscopic IPAA	Complications and quality of life outcomes	NS	*Int J Colorectal Dis*. 2021 Jul;36(7):1345‐1356
Rectal stump management	Mortality, complications, Pelvic stump dehiscence	Mortality: 1.7%, wound infection 11.3% Stump leak: 4.9%, pelvic abscess / sepsis 5.7%	*Tech Coloproctol*. 2020 Jul;24(7):671‐684
IPAA (CD vs UC)	Complications, functional outcome	CD unfavorable: pouch fistulae (OR 6.08), strictures (OR 1.82), failure, (OR 5.27) CD = UC: pouchitis	*J Crohns Colitis*. 2020 Mar 13;14(3):418‐427
IPAA	Outcomes following IPAA with and without proximal stoma diversion	Non‐diverted favorable: Anastomotic strictures (OR 0.40), pouch failures (OR 0.54), diverted favorable: Re‐operation (OR 2.51)	*Int J Colorectal Dis*. 2021 Apr;36(4):657‐669
IPAA in the elderly aged >50 years	Perioperative safety and long‐term functional success	The overall morbidity and mortality rates (30 d): 47.3% and 1.3% Functional outcomes 50‐65 vs >65 years: NS	*Colorectal Dis*. 2021 Aug;23(8):2062‐2074
Bariatric surgery	Adverse events, change in medications	Early/late adverse events: 15.9%/16.9% IBD medications: decrease 45.6%, increase 11%, no change 57.6%	*Obes Surg*. 2020 Oct;30(10):3872‐3883
Bariatric surgery	Wound infection, Clavien‐Dindo grade >II and IBD exacerbation	Wound infection (4.1%), CD grade >II (2.0%) and IBD exacerbation (4.3%). Bariatric surgery is safe in patients with IBD	*Clin Obes*. 2020 Dec;10(6):e12405
Bariatric surgery	Quality of life	Half of patients had decrease in their IBD medications after bariatric surgery	*Obes Surg*. 2020 Oct;30(10):3872‐3883
Postoperative infectious complications	IBD medications on the risk of postoperative infections within 30 d	Corticosteroids (OR 1.70), immunomodulators (OR 1.29 NS), anti‐TNF (OR 1.60), anti‐integrin (OR 1.04 NS), 5‐ASA (OR 0.76 NS)	*Cochrane Database Syst Rev*. 2020 Oct 24;10(10):CD013256
Preoperative anti‐TNF	Overall, infectious, and noninfectious postoperative complications	Use of anti‐TNF agents increases in postoperative complications: overall (OR 1.13), infectious (OR 1.44), noninfectious (OR: 1.44)	*Eur J Gastroenterol Hepatol*. 2021 Jun 1;33(6):799‐816
Vedolizumab	Overall and infectious postoperative complication rates	Overall complications: NS Infectious complications: NS	*South Med J*. 2021 Feb;114(2):98‐105
Vedolizumab (VDZ)	Postoperative complications	Overall complications (OR 1.25 NS) VDZ favorable: infection (OR 0.49) VDZ unfavorable: SSI (OR 2.97), ileus (OR 2.16), mucocutaneous separation (OR 4.69)	*Int J Colorectal Dis*. 2021 Oct;36(10):2081‐2092
Surgical treatment for ulcerative colitis			
Predict colectomy	Prognostic factors (predict colectomy)	Pediatric Ulcerative Colitis Activity Index score, hemoglobin, hematocrit, albumin, family history of UC, extraintestinal manifestations, disease extension over time	*Gastroenterology*. 2021 Jan; 160(1):378‐402.e22
Two‐stage restorative colectomy	Complication rates (modified 2‐stage, classic 2‐stage, 3‐stage approaches)	Pediatric pts: modified 2‐stage approaches leak rates higher, adult cohorts: modified 2‐stage approaches lower leak rates	*Int J Colorectal Dis*. 2020 Oct; 35(10):1817‐1830.
Surgical treatment for Crohn's disease			
Kono‐S anastomosis	Recurrence (rec.), complications	Surgical rec. 0%, endoscopic rec. 5%, ileus 3%, small bowel obstruction 4%, anastomotic leak 1%, postoperative infection 10%	*Surg Today*. 2021 Apr;51(4):493‐501
IPAA	Long‐term functional outcomes The pouch failure rate	Mean 24‐h stool frequency: 6.3 bowel movement, overall pouch failure rate:15%, no risk factors for pouch failure were identified	*Dis Colon Rectum*. 2021 Mar 1;64(3):355‐364
Surgery vs initial medical therapy	Early bowel resection (EBR) relapse rate, Newcastle‐ Ottawa and Jadad scales	EBR favorable: overall/surgical relapse (OR 0.53/0.47), requirement biologic therapy (OR 0.24), RFS, (OR 0.62), morbidity (NS)	*Int J Colorectal Dis*. 2020 Mar;35(3):501‐512
Strictureplasty (SPX)	Recurrence‐free survival SPX vs bowel resection (BR)	SPX alone increased disease recurrence than BR (HR 1.61) No difference in morbidity	*Int J Colorectal Dis*. 2020 Apr;35(4):705‐717
Stem cells therapy	The efficacy and safety for CD fistula Stem cell therapy vs placebo	Stem cell group favorable: fistula healing (61.8% vs 40.5%, OR 2.21) the treatment‐related adverse events (RR 0.58)	*Stem Cell Res Ther*. 2021 Jan 7;12(1):32
Risk of recurrence (rec.)	Risk of clinical, surgical and endoscopic (ES) rec. in positive resection margins, granulomas or plexitis	Positive resection margins: clinical/surgical /ES rec. (RR 1.26/1.87/ND), Granulomas: clinical/surgical /ES rec. (RR 1.31/1.37/NS), Plexitis: ES/clinical rec (RR: 1.31/NS)	*Clin Gastroenterol Hepatol*. 2021 Mar;19(3):451‐462
Rate of recurrence	Postoperative recurrence (POR)	TNF‐α agents (1 y) endoscopic, clinical, surgical POR: 21.7, 13.1, 3.8%, 5‐y rec. rate: endoscopic, surgical rec.:84.2%, 17.5%	*J Dig Dis*. 2021 Jul;22(7):399‐407

Fistula formation in CD is an intractable disease that is difficult to treat. The injection of allogeneic or autologous tissue is a promising new treatment for perianal fistulas in CD. Panes et al^37^ reported the efficacy and safety of allogeneic adipose‐derived mesenchymal stem cells in the treatment of complex perianal fistulas in CD. Additionally, the effects of the injection of autologous adipose tissue as a treatment modality for fistulas were reported in a cohort of CD patients in 2019.^38^ Further trials on autologous tissue implantation have been attempted for fistulas in patients with CD in phase 1 trials and RCTs. Autologous subcutaneous,^39^ adipose‐derived,^40^ and allogeneic mesenchymal stem cells,^41^ as well as bone marrow‐derived mesenchymal stromal cells,^42^ were harvested in these trials, and the results suggested that such treatments for fistulas may be effective and feasible (Table 3). In a meta‐analysis (n = 1252), the stem cell treatment group had a higher rate of fistula healing than the placebo group (61.8% vs 40.5%, OR: 2.21).^43^

The number of patients admitted for UC is increasing; however, the number of surgeries per admission is decreasing.^2^ Colonic perforation, life‐threatening bleeding, and toxic megacolon remain key indicators for emergency surgery, regardless of therapeutic advancements. Biologic agents have been used in patients with severe ulcerative colitis who are refractory to steroid therapy. Patients who are refractory to steroid therapy and infliximab are recommended for surgery.^44^ Even when control is achieved with drugs, the problem of carcinogenesis remains. This poses a significant issue, particularly since the prognosis of UC patients with colorectal cancer is worse than that of colorectal cancer patients without UC.^45^

Song et al reported that elderly onset ulcerative colitis (EOUC) was increasing. In a Korean study, the 10‐year cumulative colectomy rate was significantly higher in the EOUC than in the NEOUC group. The EOUC patients also had a higher mortality rate.^46^ In Japan, it was reported that patients with EOUC had more advanced inflammation, were more likely to be hospitalized, had greater corticosteroid use, and were more likely to require surgical treatment for UC than NEOUC patients.^47^

Ileal pouch‐anal anastomosis (IPAA) was selected for patients who required total proctocolectomy. In recent years, the number of elderly patients undergoing the said procedures for UC has increased (age >50 years). The overall 30‐day morbidity and mortality rates after surgery for elderly patients were 47.3% and 1.3%, respectively. Neither short‐ nor long‐term functional outcomes were significantly different between patients aged 50‐65 years and elderly patients (age >65 years).^48^ However, the general condition, organ function, anorectal function, and activities of daily living (ADL) were impaired in the EOUC patients.^49^ It is therefore important to select an appropriate procedure.

Proximal stoma diversion is commonly constructed when IPAA is performed. Anastomotic strictures and pouch failures have been shown to be more common in diverted patients than in non‐diverted patients, but re‐operation was more frequently required in non‐diverted patients. However, this meta‐analysis contained only one RCT; more evidence‐based research is therefore desirable to exclude selection bias.^50^ Retrospective studies showed that postoperative stoma outlet obstruction, a complication after stoma construction, occurred in 7.0%‐16.9% of IBD patents who underwent IPAA. Maximum stoma drainage volume, loop ileostomy, and body mass index (≥22.2) are risk factors for stoma outlet obstruction, and thick rectus abdominis is associated with recurrent stoma outlet obstruction.^51^, ^52^

Ileal pouch‐anal anastomosis is a safe procedure for EOUC patients; however, anorectal function and ADL should be considered when determining whether to perform the procedure. Further studies are needed to determine how to construct the diverting stoma.

### 2.5 Perioperative managements for patients with IBD

Patients with IBD are often immunosuppressed before surgery, therefore requiring careful perioperative management. A meta‐analysis examined the combined data from 68 published studies and identified the association between IBD medication and infectious complications within 30 days after surgery for IBD. Most patients were 18 years or older, and both men and women participated. The patients were divided into five groups based on their IBD medications (corticosteroids, anti‐TNFα agents, immunomodulators, anti‐integrin agents, and 5‐ASA); the patients in these groups were compared with those who were not taking medications. Total infectious complications were associated with the use of corticosteroid (OR: 1.70) and anti‐TNFα agent (OR: 1.60); however, they were not associated with the use of immunomodulators (OR: 1.29), anti‐integrin agents (OR: 1.04), or 5‐ASA (OR: 0.76).^53^ The patients treated with vedolizumab (VDZ) showed a decrease in the incidence of infectious complications (OR: 0.49), but the risks of surgical site infection (SSI) (OR: 2.97), superficial SSI (OR: 2.24), and ileus (OR: 2.16) increased. The reason for the decreased risk of infectious complications may be that VZD suppresses intestinal inflammation by inhibiting the interaction between MAdCAM‐1 and α4β7 and preventing T cells from entering the intestinal mucosal system. The suppression of intestinal inflammation by VDZ reduces postoperative infectious complications, whereas excessive immunosuppression may be the cause of postoperative SSI.^54^

Vitamin D is commonly deficient in patients with CD and the risk of CD‐related surgery increases in patients with lower serum vitamin D levels. However, the incidence of postoperative endoscopic or clinical recurrence of CD in patients who underwent ileocolonic resection was not found to be significantly different between patients who received postoperative treatment with high‐dose vitamin D and those who received placebo.^55^

In Crohn's disease, the treatment strategy emphasizes the preservation of intestinal function. Although Crohn's disease rarely leads to short bowel syndrome, some cases of this condition are unavoidable.^56^ Teduglutide, a glucagon‐like peptide‐2 (GLP‐2) analog, has been approved in Japan for the treatment of short bowel syndrome. Teduglutide promotes improved intestinal absorption function and reduces the need for long‐term intravenous support, which is difficult to manage.^57^

Pouchitis is a common complication after total proctocolectomy with IPAA, and if it persists (≥3 episodes/y) despite antibiotic treatment, is considered as chronic antibiotic refractory pouchitis (CARP). The efficacy and safety of treatment with infliximab (IFX; n = 22), adalimumab (ADA; n = 42), and vedolizumab (VDZ; n = 144) were evaluated in a meta‐analysis. Clinical improvement rates after treatment with IFX, ADA, and VDZ were 71.4%, 58.2%, and 47.9%, respectively, and the clinical remission rates were 65.7%, 31%, and 47.4%, respectively.^58^ Biologic therapy is effective in the treatment of CARP. Moreover, HBO has been reported as an effective therapy for CARP in a meta‐analysis (Tables 3 and 4).^59^

## 3 DIVERTICULITIS

### 3.1 Characteristic diverticulitis in a recent study

In the United States, the first incidence rate of diverticulitis was 2.9% between February 2015 and February 2020. The risk factors for the incidence of diverticulitis were being male, elderly (age >65 years), and Caucasian.^60^ Low physical activity, overweight and obesity,^61^ smoking, appendectomy, proton‐pump inhibitors, and non‐steroidal anti‐inflammatory drug use^62^ are other established risk factors for diverticular disease. The risk of developing such was reduced by high fiber intake. Individuals consuming fiber (30 g/d) have a 41% risk reduction compared to those with low fiber intake.^63^

The right‐ and left‐sided acute colonic diverticulitis has different characteristics. Right‐sided diverticulitis affects younger, male (OR: 1.33), and taller patients with a lower body mass index. Smoking (OR: 2.23), alcohol consumption (OR: 1.85), and comorbidity (OR: 0.21) were also associated with right‐sided diverticulitis. Further, it has a more favorable outcome with lower risk of complications, less frequent emergency surgery, recurrence, and length of hospital stay (Table 6 and 7).^64^, ^65^

**TABLE 6:** Clinical trials of diverticulitis and diverticular disease

Factor	Endpoints	Main results	Reference
Characteristics			
Right side vs left side diverticulitis	Clinical features	Right side: younger, male, tall, lower BMI, less advanced Hinchey stages, shorter hospital stays, less recurrent	*Sci Rep*. 2020 Feb 28;10(1):3754
Nonoperative treatment			
Vitamin D vs placebo	Time to diverticular disease hospitalization from randomization	Vitamin D: 1.4% and placebo: 1.5% (NS)	*Clin Nutr*. 2021 Mar;40(3):839‐843
Antibiotic treatment vs placebo	Treatment effect	Antibiotic treatment = placebo: length of hospital stay, adverse events, readmission, intervention, inflammation markers, pain	*Clin Gastroenterol Hepatol*. 2021 Mar;19(3):503‐510.e1
Surgical treatment			
Primary anastomosis (PRA) vs Hartmann's procedure (HP)	Costs and cost ‐effectiveness	PA favorable: overall costs: PAR vs HP (€20 544 vs €28 670), incremental cost‐effectiveness: €‐39 094 cost‐utility: €‐101 435	*Br J Surg*. 2020 Nov;107(12):1686‐1694
PRA vs HP	Long‐term outcomes and quality of life (QoL)	PA favorable: general QoL (EQ‐VAS), EQ‐5D index scores, PA = HP: GIQLI (intestine‐specific QOL)	*Int J Colorectal Dis*. 2021 Oct;36(10):2159‐2164
Surgery or not	QoL at 5‐year follow‐up	Surgery favorable: potential to improve quality of life	*Ann Surg*. 2020 Aug;272(2):284‐287
Damage control surgery (DCS)	Rate of stoma at discharge and at 6 mo	DCS 8.3% vs conventional treatment 57% (at discharge), DCS 0% vs conventional treatment 42% (at 6 mo)	*World J Surg*. 2020 Dec;44(12):4098‐4105
Hartmann's reversal	Factors of morbidity and mortality of Hartmann's reversal	Low albuminemia, renal failure, coronary artery disease, corticosteroids	*Sci Rep*. 2020 Feb 27;10(1):3643

**TABLE 7:** Meta‐analyses of diverticulitis and diverticular disease

Focus	Endpoints	Main results	Reference
Characteristics			
Right‐sided diverticulitis	Characteristic, comorbidity, recurrence (rec.)	Younger, male, smoking, alcohol consumption, less comorbidity, lower rec., less emergency surgery, shorter length of hospital stays	*Colorectal Dis*. 2020 Dec;22(12):1908‐1923
Left‐sided acute diverticulitis	Disease severity, risk of recurrence according to age	Young = elder: need emergency surgery or drainage, recurrence	*Eur J Gastroenterol Hepatol*. 2020 May;32(5):547‐554
Preventive medicine Fiber intake	The risk of diverticular according to dietary fiber intake, fiber subtypes	Risk reduction: 23/41/58% (Fiber intake 20/30/40 g/d). Cereal/fruit/vegetable fiber per 10 g/d (RR 0.74/0.56/0.80)	*Eur J Nutr*. 2020 Mar;59(2):421‐432
Nonoperative treatment			
Conservative treatment for uncomplicated right‐side diverticulitis	Treatment failure, ES at rec for uncomplicated right‐sided diverticulitis	Treatment failure: 2.5%, rec. rate: 10.9%, complicated diverticulitis at rec: 4.4%, emergency surgery at rec: 9.0%	*Int J Colorectal Dis*. 2021 Aug;36(8):1791‐1799
Observation vs Antibiotic treatment for uncomplicated diverticulitis	Rates of ongoing diverticulitis, rec., complicated diverticulitis, sigmoid resection	NS Observational management of uncomplicated AD is safe	*Br J Surg*. 2020 Jul;107(8):1062‐1069
Intravenous antibiotics for Right‐sided diverticulitis (Hinchey I/II)	Rec. rate, morbidity associated with rec.	Rec. rate: 12% (nonoperative management is safe and feasible) required urgent surgery at the time of first rec.: 9.9%	*Dis Colon Rectum*. 2020 Oct;63(10):1466‐1473
Nonoperative management for sigmoid complicated diverticulitis with abscess	Relapse rate at 30 d, rec. of AD	Relapse rate: 18.9%, rec. of AD: 25.5% (distant abscess: 51% vs pericolic abscess: 18%)	*Langenbecks Arch Surg*. 2020 May;405(3):277‐281
Nonoperative management for complicated diverticulitis with abscess	Treatment failure (time intervals: 1986‐2000, 2000‐2010, 2010‐)	Treatment failure rate at 90 days: 16.4%, (1986‐2000:19.2%, 2000‐2010:18.6%, 2010‐: 15.3%, NS)	*Int J Colorectal Dis*. 2021 Jul;36(7):1367‐1383
Surgical treatment			
Primary anastomosis (PRA) vs Hartmann's procedure (HP)	Mortality, morbidity, stoma reversal after surgery for Hinchey III or IV diverticulitis	PRA favorable: stoma reversal, reversal‐related morbidity, PRA = HP: mortality, morbidity, reintervention rates	*Int J Colorectal Dis*. 2020 Aug;35(8):1371‐1386
PRA vs HP	Mortality, morbidity after surgery for Hinchey III or IV diverticulitis	NS	*Arq Bras Cir Dig*. 2021 Jan 15;33(3):e1546
PRA vs HP for perforated diverticulitis with generalized peritonitis	Stoma rate, 30‐day mortality, overall mortality, major complications	PRA favorable: stoma rate, overall mortality, major complications, PRA = HP: 30‐day mortality	*Tech Coloproctol*. 2020 Jun;24(6):527‐543
Open vs laparoscopic surg. (LS) for diverticulitis with colovesical fistula	Operative time, stoma rates, complications, mortality	Open = LS: operative time, stoma rates, leakage, SSI, mortality, LS favorable: postoperative complications, length of stay	*ANZ J Surg*. 2021 Sep; 91(9):E570‐E577.
Open vs LS emergency surgery for complicated diverticulitis	Mortality, morbidity, severe complications, and reoperation rates	Open = LS: postoperative mortality, morbidity, severe complications, and reoperation rates	*Medicine*. 2020 Oct 2;99(40):e22421
Damage control surgery for complicated diverticulitis	Leakage, mortality	Major leakage: 4.7%, overall mortality: 9.2%	*Int J Colorectal Dis*. 2021 May;36(5):867‐879
Surgery for immune‐suppressed patients	Mortality	Elective surgery: immunosuppressed = immunocompetent patients, emergent surgery: immunocompetent patients favorable	*Am J Surg*. 2021 Jan;221(1):72‐85

### 3.2 Treatment approach for diverticulitis

Patients with uncomplicated acute diverticulitis are commonly treated with antibiotics; however, in recent RCTs, this has been omitted for acute uncomplicated diverticulitis, which is characterized by elevated body temperature, inflammatory parameters, except for sepsis, and any sign of complications, such as abscess, free air, or fistula on computed tomography. Moreover, an earlier study demonstrated that the median hospital stay duration, adverse events, and hospital readmission were not significantly different during antibiotic treatment and observation.^66^ In a 1‐year follow‐up of patients with uncomplicated diverticulitis, the rates of ongoing, recurrent, and complicated diverticulitis and undergoing sigmoid resection were not significantly different during antibiotic treatment and observation.^67^ These results provide evidence for the omission of antibiotics in patients with uncomplicated acute diverticulitis.

In cases of recurrent diverticulitis or persistent symptoms, the choice between surgery and conservative treatment is an important concern. The failure rates of nonoperative management for acute diverticulitis with complicated abscesses is 16.4%, and the failure rates have not significantly decreased in the last 30 years.^68^ The failure rate of percutaneous drainage as a nonoperative management for patients with pelvic abscess was three times higher than that for pericolic abscesses.^68^, ^69^ Hence, surgical treatment should be considered for abscesses in areas distant from colonic diverticulitis.

The aim of diverticulitis surgery is to treat acute inflammation and symptoms, and improve quality of life (QOL). A previous study compared elective sigmoid resection and conservative management in patients who had ongoing abdominal complaints for >3 months and/or frequently recurring left‐sided diverticulitis of >2 episodes in 2 years. The Gastrointestinal Quality of Life Index of the patients after sigmoid resection was higher than that after conservative treatment.^70^ Colonic resection for recurrent diverticulitis improved QOL, and these data are helpful in determining the indications for bowel resection.

### 3.3 Surgical procedure for diverticulitis

The major concerns of acute diverticulitis surgery are associated with bowel resection. These factors were analyzed in meta‐analyses and RCTs (Table 6 and 7).

Primary anastomosis (PRA) and nonrestorative resection (NRR), which are defined as end colostomy or diverting transverse colostomy without resection, respectively, were selected as emergency surgeries. The morbidity rates after PRA did not differ from those after NRR; however, postoperative morbidity rates of stoma closure surgery were significantly lower in PRA (12%) than in NRR (27.2%) (OR: 0.31). The non‐reversal rate of stoma in PRA (16%) was lower than that in NRR (35.5%) (OR: 0.37).^71^ Moreover, PRA was associated with better short‐ and long‐term outcomes than NRR.

Further, it is important to establish whether PRA or Hartmann's procedure (HP) should be selected when performing colonic resection for acute diverticulitis. PRA has favorable rates of stoma reversal (PRA, 80.3%; HP, 62.1%; OR, 2.62) and reversal‐related morbidity (PRA, 11.9%; HP, 27%; OR, 0.33). The mortality (PRA 5%, HP 6.4%, OR: 0.83, n.s.), morbidity (PRA 50.6%, HP 49.5%, OR: 0.99, n.s.), and reintervention rates (PRA 7.4%, HP 7.5%, OR: 0.90, n.s.) did not differ between PRA and HP.^72^ Moreover, the overall mean costs per patient were also lower for PRA (€20 544) than for HP (€28 670), with a mean difference of €8126.^73^ Based on these data, PRA should be considered, if possible.

In a meta‐analysis, emergency laparoscopic surgery for colonic diverticulitis had a lower morbidity rate than open surgery, though the rates of postoperative mortality, severe complications, and reoperation did not differ. However, this meta‐analysis was based on both RCTs and retrospective studies. A greater number of high‐quality RCTs are necessary to compare laparoscopic surgery and open surgery.^74^ Laparoscopic surgery is classified into two types; laparoscopic primary resection and laparoscopic lavage without primary resection. Laparoscopic peritoneal lavage is associated with higher morbidity than laparoscopic primary resection.^75^

Immunosuppressed patients with diverticular disease have an increased risk of developing complicated diverticulitis. The mortality and morbidity rates of immunosuppressed patients were higher than those of immunocompetent patients for emergent surgery (RR: 1.91 and RR: 2.18, respectively), but not for elective surgery (RR: 1.70 and RR: 1.40, respectively).^76^ Elective surgery may be planned for immunosuppressed patients with diverticulitis according to a meta‐analysis.

### 3.4 Recurrence after diverticulitis surgery

A time‐to‐event analysis for recurrence‐ and colostomy‐free survival was performed using a large retrospective cohort. Of the patients with uncomplicated diverticulitis treated with non‐operative methods, 19% underwent elective surgery and 81% were treated medically for recurrent uncomplicated diverticulitis after initial therapy. Patients who underwent elective surgery were associated with lower rates of recurrence than those treated with medical therapy (15% vs 61% at 5 years, OR: 0.17). The rate of colostomy after elective surgery (1.8%) was lower than that after medical therapy (2.3%) at 5 years (OR: 2.3).^77^ The recurrence rate of diverticulitis was reported to be 5.8% in a meta‐analysis. Six factors related to recurrence after bowel resection with diverticulitis were identified: younger age and irritable bowel syndrome (preoperative); anastomotic level and uncomplicated recurrent diverticulitis (operative); absence of active diverticulitis on pathology and persistence of postoperative pain (postoperative). According to the results of this study, elective surgery prevents diverticulitis recurrence or colostomy risk.^78^

## 4 FUTURE PERSPECTIVE AND SUMMARY

In this review, we present key articles on clinical trials and meta‐analyses of IBD and diverticulitis from 2020 to 2021. The development of new drugs for IBD is remarkable, and treatment strategies using multiple agents and various techniques are required. It is necessary for surgeons to have a deep understanding of the surgical procedure and perioperative management, as well as the impact of new drugs. In terms of the surgical procedure for CD, it is important to select an optimal procedure that preserves bowel function, minimizes recurrence, and reduces complications. The usefulness of autologous cell transplantation for fistulae on CD has been verified, and it may be clinically applied in the future. It is also important to enhance knowledge on perioperative management associated with IPAA in total proctocolectomy for IBD.

The incidence of diverticulitis is increasing, and more patients are expected to require surgical treatment in the future. According to recent reports, antimicrobial therapy is unnecessary for uncomplicated diverticulitis. Regarding surgical procedures for diverticulitis, both bowel resection and anastomosis are associated with favorable short‐term outcomes, higher stoma closure rate, and more reasonable medical costs than HP. The risk factors for the recurrence of diverticulitis are summarized, and these data support the optimal management for postoperative diverticulitis patients. This review consolidates the available knowledge and improves the quality of surgical procedures and perioperative management in treating IBD and diverticulitis.

## DISCLOSURE

Funding: No funding was received for this study.

Conflict of Interest: The authors declare no conflict of interest for this article.

## Supporting information

Table S1
