# Supplementary material for: Essential updates 2020/2021: Colorectal diseases (benign)—Current topics in the surgical and medical treatment of benign colorectal diseases
Source: Ann Gastroenterol Surg. 2022 Jan 25;6(3):321–35. doi: 10.1002/ags3.12548 (PMC9130914; doi:10.1002/ags3.12548)
Supplement: Supplementary file 1 — Table S1 [file AGS3-6-321-s001.docx]

**Supplementary Table S1.** Approval status of novel agents for inflammatory bowel disease.

| Agents | FDA Approval status | Europe Approval status | Disease | Japan Approval status | Disease |
| --- | --- | --- | --- | --- | --- |
| Upadacitinib | Yes, Aug-2019 | Yes, Dec-2019 | Rheumatoid Arthritis in Adults | Yes, Apr -2020 | Rheumatoid Arthritis in Adults psoriatic arthritis atopic dermatitis |
| Ozanimod | Yes, Mar-2020 | Yes, May-2020 | Multiple sclerosis in adults | No |  |
| Mongersen | No | No |  | No |  |
| Etrasimod | No | No |  | No |  |
| Vedolizumab | Yes, May-2014 | Yes, May-2014 | Acute ulcerative colitis Crohn’s disease | Yes, Jun-2018 | Acute ulcerative colitis Crohn’s disease |
| Cobitolimod | No | No |  | No |  |
